# Is restlessness best understood as a process? Reflecting on four boys’ restlessness during music therapy in kindergarten

**DOI:** 10.1080/17482631.2017.1298266

**Published:** 2017-05-22

**Authors:** Anna Helle-Valle, Per-Einar Binder, Norman Anderssen, Brynjulf Stige

**Affiliations:** ^a^ Department of Psychosocial Science, Faculty of Psychology, University of Bergen, Bergen, Norway; ^b^ Department of Clinical Psychology, Faculty of Psychology, University of Bergen, Bergen, Norway; ^c^ Grieg Academy – Department of Music, Faculty of Fine Art, Music and Design, University of Bergen, Bergen, Norway

**Keywords:** ADHD, restlessness, process, vitality, children

## Abstract

ADHD can be considered an internationally recognized framework for understanding children’s restlessness. In this context, children’s restlessness is understood as a symptom of neurodevelopmental disorder. However, there are other possible understandings of children’s restlessness. In this article, we explore four boys’ collaborative and creative process as it is described and understood by three adults. The process is framed by a community music therapy project in a Norwegian kindergarten, and we describe four interrelated phases of this process: *Exploring musical vitality and cooperation, Consolidating positions, Performing together*, and *Discovering ripple effects*. We discuss these results in relation to seven qualities central to a community music therapy approach: participation, resource orientation, ecology, performance, activism, reflexivity and ethics. We argue that in contrast to a diagnostic approach that entails a focus on individual problems, a community music therapy approach can shed light on adult and systemic contributions to children’s restlessness.

ADHD was first included in the *Diagnostic and Statistical Manual* (American Psychiatric Association) in 1980, and the number of children diagnosed with ADHD has since dramatically increased in both the USA and globally (Neufeld & Foy, 2006; Polanczyk, Lima, Horta, Biederman, & Rohde, 2007; Polanczyk, Willcutt, Salum, Kieling, & Rohde, 2014; Rowland, Lesesne, & Abramowitz, 2002). Given the presence of clients diagnosed with ADHD in health care, education and everyday life across cultures, this diagnosis can be considered an internationally recognized framework for interpreting and handling children’s restlessness (Barkley, 1997; Rowland et al., 2002; Subcommittee on Attention-Deficit/Hyperactivity & Committee on Quality, 2001; Ullebø, 2010). Within mainstream research on ADHD, children’s restlessness is understood as a symptom of neurodevelopmental pathology. However, children’s restlessness can be understood in different ways depending on the epistemological and ontological stance of the researcher.

Post-positivism and social constructionism are two well-established research traditions within the research field of ADHD, and these carry with them assumptions about epistemology and ontology. According to a post-positivist research tradition, theories are seen as human made linkages between single-sense data. According to social constructionism on the other hand, all knowledge is linked to social constructions and should not rise too high above these (Alvesson & Sköldberg, 2000). This means that social constructionism entails taking a critical stance towards taken-for-granted knowledge, exploring historical and cultural aspects of this knowledge, and acknowledging the close relation between knowledge and social action (Burr, 2015). The individual researcher might express such epistemological and ontological differences though choice of methods or words, but they might not be fully aware of, or reflect on, their own position. In this article, we write from a social constructionist stance.

Researchers who adhere to a social constructivist epistemology are often labelled “critical” within mainstream research on ADHD. Such research can shed light on how adults and experts rely on socially constructed practices and knowledge when judging whether children’s behaviours should be understood as symptoms of ADHD (Moncrieff & Timimi, 2013; Timimi, 2005; Timimi & Taylor, 2004). Another example is how in the case of mothering practices, ADHD has the function on shifting blame from the mother to the child’s brain, but does little to actually pierce oppressive mothering ideals (Singh, 2004).

Mainstream research on ADHD is often carried out using quantitative methodologies and downplays the role of social constructions. One example of this is the consensus statement signed by a consortium of international studies, in which statements by doctors or researchers that question whether ADHD is a real medical condition are referred to as “attempts at balance [that] give the public the impression that there is a substantial scientific disagreement over whether ADHD is a real medical condition. In fact, there is no such disagreement” (Barkley, 2002, p. 89).

From a social constructivist perspective, rather than being an objectively measurable, context-independent and stable symptom of neurological disease, children’s restlessness can be understood as an integrated aspect of a *process* that heavily relies on adult participation and judgement. In this case, restlessness can be recognized as a *form of vitality* that is experienced and assessed by more or less invested adults. *Forms of vitality* are defined as the manifestation of life as expressed through movement, experienced and understood by a human being (Stern, 2010).

As a form of vitality, restlessness can be included as an aspect of “normal development”. From this perspective, children have the right to experience restlessness as a natural and safe aspect of vital interaction (Johns, 2012). Children’s natural musicality is arguably central to the development of cooperative awareness (Trevarthen, 2000), and musical experiences and musical relationships can therefore be used as dynamic forces of change. This claim is based on the increasing evidence suggesting that human protomusicality is the basis of human companionship (Malloch & Trevarthen, 2009). According to the theory of *communicative musicality*, all humans have an innate capacity to sympathize with the rhythmic and melodic movements of body and voice, as demonstrated by pre-linguistic infants communicating with adults. Malloch and Trevarthen (2009) argue that children are born with a uniquely human motivation for gestural communication, a talent that later in life may be cultivated into general communication skills as well as conventional musical abilities. In music therapy, such processes are facilitated by the therapist with the aim of optimizing the client’s health (Bruscia, 2014).

The explicit focus of music therapy is to improve the health of the client. However, “health” can be understood in different ways. Within music therapy, there are at least two approaches to health: it can be understood with an emphasis on individual problems and function, or as a relational concept that emerges between the person and his or her surroundings. Understanding health as individual function and symptoms is potentially a good fit with an individual and problem oriented approach to children’s restlessness, like ADHD. Indeed, music therapy has been used to improve children’s academic performances (Camilleri, 2000; Chong & Kim, 2010; Steele, Vaughan, & Dolan, 1976), reduce their restless behaviours (Gold, Voracek, & Wigram, 2004; McCarty, McElfresh, Rice, & Wilson, 1978; Sausser & Waller, 2006), and treat ADHD (Miller, 2007, 2011).

The reflections in this article rely on a relational concept of health, often referred to as the *interpersonal approach* (Kaslow, 1996; Kiesler, 1991). In music therapy traditions that rely on a relational concept of health, practice is commonly focused on the relationship between the person and his or her surroundings (Stige & Aarø, 2012). Health is in this sense closely related to an individual’s possibilities for action (Ruud, 1998). This means that health is not only about treating individual symptoms or about improving individual function. More broadly, it is about working towards facilitating increased possibilities for action for persons who are marginalized by mainstream practices. Within music therapy, music is understood as an activity—*musicking* (Small, 1998)—that facilitates participation in the widest sense. In relation to children’s restlessness, music therapy can allow for a broad range of participatory behaviours. Music therapy can also support participation through for instance increasing motivation or recognizing and facilitating restlessness as a valid form of nonverbal communication.

In this article, we will use case studies from music therapy to reflect on how children’s restlessness has been described and handled, and how it can be understood. We will first present a review of music therapy case studies in which the author(s) have used the terms “restlessness”, “hyperactivity”, “impulsivity”, “hyperkinetic”, or “ADHD”. We have been inspired by a critical interpretive approach as described by McFerran, Hense, Medcalf, Murphy, and Fairchild (2016). This approach outlines a systematic and iterative methodology for approaching, gathering, interrogating and synthesizing relevant literature. It allows the researcher to reflect on intuitive and emotional reactions to the literature, and facilitates reflections about contextual and historical aspects of research. One example of this is how the need for a diagnostic approach is affected by local problems and needs, like problems in the classroom and home setting, but also by global epistemological and political trends, like the dominating position of post-positivism and the global rise in children diagnosed with ADHD.

We present the results from the reviewed case studies based on geographical region in order to highlight context. After the reflexive summary, we will present and discuss a case study from a community music therapy project in a Norwegian kindergarten.

## Case descriptions from USA and Canada

Aigen (1991) describes Will, a musically and intellectually gifted eight year old boy, who was brought to therapy for fighting in school. Aigen describes the therapeutic process where he accesses Will’s fantasy world through music. He describes how Will at a point during therapy stops wanting to play music, and how he increasingly gets into fights at school. Aigen explores the topic of violence and aggression with Will. He is left with the impression that Will plays the role of a scapegoat at school, considers himself a “bad boy”, and that he feels forced by his mother to attend many after-school activities. This leads Aigen to set up a meeting with Will’s mother and her therapist, and Will has the opportunity to share his perspectives. Will’s prize for attending this session is a joint musical improvisation with his mother and her therapist. Will enjoys this so much that he requests they meet again as a group. After a break in therapy due to summer holidays, Aigen describes Will as having a rekindled love for music, and as more mature. Aigen reflects on how Will’s problems can be related to unfortunate circumstances like a difficult family situation, and pressure to perform both in school and in various after-school activities.

Herman (1991) describes Robbie, a likable little 12-year-old who has lived in 12 foster homes and two treatment centres before being admitted to a children’s psychiatric hospital. Robbie considers himself *the boy who nobody wanted*, and struggles with hyperactive behaviours and lack of concentration. Herman describes his process as going through five stages: opening up, gaining expressive freedom, enjoying self-expression, learning structure, and being himself with others.

Hibben (1991) describes a special education group of children from 6 to 8 years, displaying destructive and disruptive behaviours associated with ADHD such as excessive activity, interruptive talking, and physical aggression. Some are medicated with psychostimulant, anti-epileptic or anti-depressant drugs. Hibben describes three stages in the music therapy process: a pre-affiliation stage where the children vacillate between approach and avoidance, a power and control stage where the children jockey for power and status, and an intimacy stage where the children begin to practise new behaviours and show their needs. Hibben describes how music helps the group towards better cohesion through providing them with both boundaries and inspiration.

## Case descriptions from New Zealand and Australia

Rickson (2002) describes a 12-year-old Maori boy, Adam, who is presented as likable, humorous and with practical skills. Adam is diagnosed with ADHD, he has reduced intellectual capacity and lives with a family with low socio-economic status. Adam’s positive interactions increase somewhat and his negative interactions decrease very much as a result of music therapy. Rickson reflects on how music therapy has presented Adam with a joyful and more constructive way of participating, and how it has created an opportunity for him to recognize himself as a skilled musical person.

In 2003, Rickson describes the case of a 12-year-old boy, John, who has been removed from his single parent home because of severe neglect. He has several diagnoses, including ADHD, and rejects taking medication. The very challenging treatment process changes and broadens Rickson’s therapeutic approach. Music is generally considered the central element in music therapy. However, because of John’s initial strong rejection of music and due to his history of abuse and neglect, Rickson allows music not to be the focus all the time. Instead, she focuses on creating a holding environment for John through silence, physical touch and short music interactions. She reflects on John’s rejection and protests as a projective re-enactment of the destructive relationship he had to his mother, and how the process of constructing a new relationship and experiencing hope might be highly ambivalent. Gradually, the music therapy process reveals John as a sensitive musician and skilled performer, and leads to John performing in the local school operetta.

McFerran (2009) describes Ben, a 12-year-old boy diagnosed with ADHD and mild intellectual disability. He recently stopped taking medication, and is referred to music therapy for aggressive behaviours in the classroom. Ben’s unrealistic evaluation of his own musical skills leads to enthusiastic participation. Taking Ritalin changes his evaluation and makes it more realistic, but stops Ben from enjoying his own music. Ben shows a need to control the therapist, but as he becomes more empowered in the music therapy setting, he also becomes a more generous participant. Ben struggles with tension that arises as he moves from music therapy to the controlled classroom setting. Despite continued difficulties in the classroom and playground setting, music therapy continues to be an empowering setting for Ben. The music therapy sessions, which are loosely structured but highly facilitated by a supportive and active music therapist, help Ben experience himself as calm, more in control of his behaviour, as more cooperative and as someone who can have fun.

## Case descriptions from UK, Norway and Finland

Johns (2012) discusses how acknowledging children’s vitality can facilitate access to children’s life worlds and create therapeutic change. She presents findings from a therapy process with a traumatized girl, and argues why children should be able to explore restlessness as a natural and non-threatening dimension of vital expression.

Hakomäki (2012) presents a collaborative research project with a 14-year-old boy, Nick, who reflects on his own therapeutic process between the ages of 7 and 9. Nick was sent to music therapy three years after losing his brother. At that time, he longed to die and struggled with restlessness in the classroom. The collaborative retrospective reflection regarding Nick’s recovery process is prompted by narrative pieces of music. Hakomäki reflects on how she co-creates a meaningful narrative with Nick, and sees this as a process of reconstructing meaning after losing a loved one. She argues that many of our constructions of reality are impossible to verbalize, but that they might find an expression through music.

## A reflexive summary and the focus of this article

The case studies presented here illustrate how restlessness has been dealt with in music therapy and related to children’s problems and potentials. Many of the clients described here are sent to music therapy with the aim of facilitating better function in the classroom situation or in everyday life.

It is striking how often the children in these case studies are neglected or abused boys brought to music therapy for aggressive behaviours. From a constructionist perspective on ADHD, children growing up in “Western” societies are understood on the basis of a polarized perspective on childhood: the victimized and innocent child who needs rescuing, and the impulsive, aggressive juvenile delinquent from which society needs protection (Timimi, 2005). A majority of the case descriptions tell the story of boys who are presented as delinquents in the classroom context and victims in relation to their family situation. The role of the music therapist seems to be to relieve, remove, or reduce the expression of pain and tension. In contrast to the other case descriptions, the publications from Norway (Johns, 2012) and Finland (Hakomäki, 2012) emphasize the victim pole, and do not present the child as delinquent. The approach described by Hakomäki (2012) is the most explicitly collaborative of all the studies.

In the case studies we have reviewed, the primary goal seems to be to facilitate change in the child. Cultural or systemic changes are rarely suggested, but examples include focusing on children’s emotional needs and quality of life instead of behavioural change (McFerran, 2009), understanding children’s restless behaviour as a natural expression that should be allowed (Johns, 2012), and requesting qualitative inquiries to increase the clinical relevance of research (Rickson & McFerran, 2007). Initiatives that aim at changing the systems or culture that surround the child depend on understandings that include system- or culture-oriented perspectives. Such perspectives are central to the family of practices that since the early 2000s has been referred to as *community music therapy* (Pavlicevic & Ansdell, 2004; Stige, Ansdell, Elefant, & Pavlicevic, 2010). In community music therapy, there is a strong focus on how individuals participate in a particular community, and the community welcomes and facilitates participation. One example of this is how persons with long-term mental health problems participate in a music group that allows them to negotiate and perform *belonging* within the group and in relation to society at large (Ansdell, 2010). Community music therapy practice is driven by ethical reflection and activism (Stige & Aarø, 2012). In line with this approach, the overall aim of this article is to promote a reflexive stance in relation to children’s restlessness. Consequently, our research question is: How can children’s restlessness be understood as a process? As research from community music therapy outlines particular process-related qualities, we will use a case from community music therapy to reflect on this question.

## Method

The data presented in this article were collected during a community music therapy project in a Norwegian kindergarten. In Norway, most children start kindergarten at the age of 1 and attend kindergarten until they are 5 or 6, as this is when they start school.

### Participants

Thirteen boys and girls between ages 5 and 6 participated in the project. Participation was determined by whether or not the child came to kindergarten that day. We chose to focus on a group of four boys because, according to the educational leaders, restlessness seemed to arise when these four boys got together. We will refer to the four boys as “Paul”, “John”, “George” and “Ryan”. The aim of this study is not to determine whether these four boys caused the restlessness, or if they were more restless than the other nine children who participated in the project. We aim to explore, describe and reflect on how restlessness can be understood as a process, with a particular focus on four boys who are understood as restless by the surrounding adults.

### Facilitation and supervision

Two music therapy students facilitated the music therapy project. We call them “Marsha” and “Brian”. The first author of this article, referred to as PI, observed and participated in the music therapy project, and supervised the students. Two music therapists employed by the Educational and Psychological Counselling Service also supervised the students. Two educational leaders participated in the music therapy sessions and were involved in planning and facilitating the project through daily meetings. We call them “Klara” and “Vera”.

### Outline of the community music therapy project

In order to meet the children and familiarize ourselves with the setting, the two music therapy students and the PI visited the kindergarten two weeks before the music therapy project officially started. During the four weeks the project lasted, the students and the PI spent three days a week in the kindergarten, each visit lasting about three hours. These days normally started and ended with a meeting where the students, the educational leaders, and the PI reflected on the progression of the project. The music therapist supervisors participated in these meetings in the beginning and towards the end of the project.

Each project day began and ended with a semi-structured music therapy group session. In order to familiarize the children with the music therapy context and facilitate and welcome their motivation, interests and contributions, the students used both structured music games and improvisation. As the children grew more familiar with the context and as their participation became more pronounced, the students relied less on structured play and improvised more. The children were also engaged through drawing, playing, and dancing. The pieces for the final performance were created in cycles: the children’s contributions and expressions were interpreted and restructured by the music therapy students and brought back to the children, who then reacted to and commented on the work. These cycles were repeated until each child had a piece in the performance that they felt comfortable with, and proud of.

Throughout the project the students were urged to be mindful of the children’s boundaries, and they did their best to avoid forcing the children into participating in a way that was too revealing or made them uncomfortable (Aasgaard, 2005). The project ended with two performances that consisted of eight pieces that included unique contributions from all the children. The students, the educational leaders, and the music therapy supervisors participated on stage during the performances, while the PI video recorded the performances.

The first performance took place in the room used for the music sessions in the kindergarten. The children’s families, and staff members from the local pedagogical resource centre, and from the local child and youth mental health services were invited. The second performance took place in the gymnasium in the local school. A group of children from the kindergarten and their teachers, the first graders and their teachers, the school principal and a few parents attended this second performance. One of the music therapists had prepared bags with Easter candy, which the principal handed out after a short speech in which she commented on the children’s performances and welcomed them to school after the summer holidays.

Three weeks after the end of the project, the PI returned to the kindergarten to interview the children about their perspectives on the process. One week later the students returned to show the children and educational leaders a recording of one of the performances, and to say goodbye to the children. Three months later, the PI returned to the kindergarten to give each child a copy of the performance on DVD, and a printed version of the performance programme listing the different pieces with credits to the children who co-created these. After this visit, the PI had a final meeting with the kindergarten teachers where they read and reflected on the transcripts of the interviews with the children, and reflected on the process of the project.

### Data collection

Data collection was collaborative and multi-modal; the students and the PI kept research diaries throughout the project. The diaries were structured as daily field notes that provided a chronological overview of what had happened that day. These descriptions also contained lines of personal reflections. These personal reflections did not have a separate column or space in the diaries, as we did not believe there could be a clear line between observations that often are regarded as more objective, and personal reflections that are regarded as more subjective. However, the students and PI were encouraged to notice when they reflected on the process in a less descriptive and more normative way.

The PI also videotaped the two performances, and interviewed the children and the educational leaders. The results from these interviews are not included in the analysis in this article, but in general the tendency was that the educational leaders expressed both pride and frustration with the process. The children mainly expressed satisfaction and enthusiasm regarding the process, but some also commented that it was challenging or boring. Both the educational leaders and the children wished for more musical activities in kindergarten in general, but the educational leaders pointed to practical challenges and lack of resources. We draw on all the data collected during our analysis, but as we were interested in describing restlessness as a process and because of space, we have chosen to focus on data from the three research diaries.

### Ethical considerations

Ethical approval was obtained from the Regional Committee for Medical and Health Research Ethics. Parents were given information about the background, intent, outline and potential risk of the study, and were informed about the confidential and voluntary nature of the study. For ethical reasons, informed consent sheets were distributed and collected by the kindergarten staff. All parents signed the agreement. All persons presented in this article have been anonymized. However, those who participated in the project, or watched the performances, might be able to indirectly identify the persons described. We have done our best to analyse and discuss the results in a respectful manner.

As described above, we were mindful of the children’s boundaries throughout the project, and did our best to engage them and make the process personal and motivating without being revealing or invading. The fact that we chose to focus on the four boys who were sometimes described as restless, presented us with an ethical challenge. In one sense, we think that gaining knowledge about restlessness as a process with an emphasis on resources and vitality can help counteract an exaggerated focus on problems and the individual child. Within the ADHD paradigm, children’s restlessness is seen as an individual problem and a stable phenomenon (see i.e. Barkley, 1997). None of the children in this study had been diagnosed with ADHD. However, we have chosen to relate our study to this diagnosis for two reasons. First, the behaviours displayed by the boys when they got together could be described as hyperactive and impulsive. Second, there is a need for more knowledge about how and when restlessness becomes a problem that needs to be assessed. In our case, the educational leaders described how they have struggled to handle the restlessness displayed by this group of boys, and they reflected on how this would play out as the boys started school. A lack of knowledge about children’s restlessness as a process is reflected in the claim that there are no known strategies for preventing ADHD (National Institutes of Health Consensus Development Conference Report, 2000). There is a need for more knowledge about restlessness *before* it becomes an issue of referral.

An overarching ethical aim should be to protect children’s vitality and facilitate self-worth and positive self-regard. Some children diagnosed with ADHD go through severe and unnoticed struggles with identity and vitality (Olsvold, 2012). Regarding restlessness as a process instead of a stable, individual disorder is in itself an ethical statement, as it points to the contributions of adults and the importance of context. We reflect on how children’s restlessness can be understood as challenging, but also as a force of vitality and creativity in music therapy. This has implications for the mainstream clinical approach to restlessness, and could inspire parents, schools and health care systems to create spaces and relations that recognize children’s restlessness as a potentially constructive force, and as an expression of their vitality and agency.

### Analysis

Our over-arching analytical approach is reflexivity, understood as a form of reflective research that involves reflection on several levels or that is directed at several themes (Alvesson & Sköldberg, 2000). We have reflected on the relations between two main themes in this article. The first theme is how restlessness can be understood from an individual- and problem-oriented perspective, hereunder ADHD. The other theme is how children’s restlessness can be understood from a perspective oriented towards participation, resources and context, like in research on community music therapy.

Our concrete analytical approach in this article is case study. A case study is an in-depth, intensive and focused exploration of a phenomenon (Willig, 2008) that facilitates reflection and deeper understanding (Stake, 1994). In this article, the case is a situated, musical and collaborative *process* during community music therapy in which we particularly focus on four boys. Furthermore, as our overarching approach is reflexivity, we have chosen to reflect on how this process was situated and co-created by the other adults and children involved.

The analysis was performed in the following steps. (1) We first immersed ourselves in the material gathered to have an overview of possible cases to follow. This was done by reading and discussing the research diaries, the interviews and by watching and discussing the videotaped performances. (2) To narrow the focus, we chose to concentrate on the process of the four boys as it was described in the three research diaries. (3) The first author created a document that contained all the diary excerpts in which the four boys were described. These excerpts were kept in chronological order and anonymized. (4) After discussing these results, we chose to focus on episodes that were judged as relevant in relation to restlessness and the process as a whole. (5) These shorter excerpts were then re-read and conceptualized as phases. The phases were named, discussed and revised. (6) A first draft of the article was sent to the educational leaders, the music therapists and the students. There were few critical comments, and overall little feedback on the text. However, one of the music therapists emphasized the importance of balancing a focus on problems and resources, and of maintaining a focus on resources throughout the text. She remembered how the four boys had been a focus also during the project, and supported the choice of making them the focus of the case study.

## Results

We present the results as four overlapping phases: *Exploring musical vitality and cooperation, Consolidating positions, Performing together*, and *Discovering ripple effects*. By presenting our results as phases, we were able to show how the restlessness changed and evolved as the boys first explored their musicality, then consolidated their roles and finally performed together. A quick name-count in the research diaries reveal how two of the boys are especially noticed: Paul’s name appears 72 times, which is the most frequently of all the 13 children. John is mentioned 66 times. This might indicate that they demanded attention in the moment, that they for some other reason were *paid* extra attention to, or that they had a lot to offer during the process which made them central contributors to the process.

The phase *Exploring musical vitality and cooperation* lasted the two first weeks of the project, and was the longest and perhaps most challenging phase in relation to restlessness.

### Exploring musical vitality and cooperation

*Diary of Principal Investigator (PI) Week 1, Day 1*: The three noisy boys went into a different room, and Klara, the educational leader, went with them. She joined their dance, and they started showing her that they could breakdance. (…) The fact that they can breakdance—we can use that in the performance.

The PI describes the boys’ restless behaviour as “noisy”. This might have been influenced by the educational leaders, who had struggled with handling this constellation of boys for several years. Still, the educational leader Klara was determined to meet the children in a playful way, and by participating with the boys on their own terms, she helped uncover their resources.

*Diary of PI Week 1, Day 2*: George, Paul and John fool around a lot. (…) After a little while Brian got them down on the floor and made a song about the things they told him. They wanted to make a song about being good friends: that they rang the neighbours’ door bell, that they couldn’t go into the forest because they were worried about the “old woman with the staff”^1^, and that they would go to the kiosk to buy waffles and ice cream.

*Brian’s Diary Week 1, Day 2*: Paul is still as eager as before in terms of playing music and playing games. (…) It seems that the children do not consider us as adults that must obey the rules of kindergarten. Paul and John went down the stairs during hide and seek, and when I found them they screamed quite loudly and created a lot of unrest for the rest of the group down there. Afterwards Paul took an apple without asking the adults if he could. This issue was quickly attended to by Vera, the educational leader, and she said that it was unusual for Paul to do something like this.

The PI describes the boys as “fooling around” and Brian refers to Paul as “eager”. Through musical improvisation and play, the boys’ interests and personal characteristics emerge. Music therapy becomes a space where change can happen, and where a new sense of community and set of rules has to be formed. The music therapy supervisors claimed it “had to get worse before it could get better”. The educational leaders, however, feared that the music therapy indirectly facilitated a process that would spiral some of the children into anti-social patterns of behaviour. Assigning responsibility for structuring the children was challenging. The students were responsible for their own practicum placement process, but had little experience. Their supervisors were experienced, but not always present, and also wanted to challenge the students. The PI was responsible for the research project, but was a participant observer. Negotiating structure, leadership and responsibility was to some degree delegated to the children:

*Brian’s Diary Week 1, Day 3*: We introduced a microphone to the children. It was mostly unstructured, noisy and chaotic, as Paul and John wanted to fool around with the microphone and did not let the others have a go.

Paul and John’s behaviour is described as “fooling around”. Their behaviour could be described as “inobedience”. It could also be called “exploration”, but this might not communicate the feeling of restlessness and frustration that “fooling around” seems to do. At the end of the first project week, a well-functioning musical community is still a work in progress.

*Brian’s Diary Week 2, Day 1*: We had a session with Paul and George, as John and Ryan were missing. The session was a bit amputated, but Paul seemed activated like before. George contributed with many ideas regarding songs they could sing and dance to, and we are going to use *Thriller*. We played this song a couple of times to find out what they could sing about. Paul and George quickly came up with suggestions about what was to be sung about them, but said “we have to wait with the lyrics for John and Ryan because they are not here”.

The absence of John and Ryan seems to create a different space for Paul and George as they engage in creative collaboration. The musical improvisation reveals a strong sense of community between the boys. John and George seem to want to protect John’s and Ryan’s right to tell their own story. Paul is noticed as being “activated” and motivated.

*Marsha’s Diary Week 2, Day 2*: George, Paul, John and Ryan were really out of hand today. A true contrast to the session just before with the two girls. The four of them came in and were not prepared to follow instructions at all. No matter how much we tried, they were all over the place. Especially John and Paul. John was very concerned with deciding that the others should do as he said. Paul just wanted to climb up on the table and play with the instruments. The only one who took instructions and showed some interest for the things we had been doing on Monday, was George. He lit up the second we started talking about *Thriller*. Despite our repeated initiatives to make games or try to invite them into things, things just never fell into place. We did not scold them, and finally we just said that they had spent their time and that we had to switch groups. That made them a bit sour and dejected, and they said it had been a boring session because they didn’t have time to play. (…) Paul and John, who had been very restless during their own session, were very cuddly and wanted to sit on Vera’s lap during the goodbye song.

It seems as if the students’ loose structure allows the children to experience the consequences of their own restlessness in terms of lack of focused attention and cooperation. According to the boys this was experienced as “boring”. In the Norwegian language, “boring” can be taken to mean “too little stimulation”, but also “sad” or “disappointing”. Their choice of words points to the efforts of the students and the situation that occurred, and deflects attention from their own contribution. This points to a paradox: in one way they experience the results of their own lack of attention and cooperation, in another way they avoid experiencing these consequences by pointing to the contributions of the students. The contrast between their unproductive restlessness and their subsequent need for care, rest and physical closeness is moving. It gives depth to the characters that so far have been described in terms of their restlessness, capability and independence.

### Consolidating positions

*Brian’s Diary Week 3, Day 1*: We started with John, Paul, George and Ryan. John was still a bit down today. He was withdrawn during the run through of *Thriller* and was clearly bothered by something. The other boys worked very well together and worked really hard during their short session. Everyone made suggestions as we played and found out what they wanted to do. (…) During the refrain when we sing all their names, they put on a bit of an extra show. A great start to a great day.

Paul, George and Ryan are motivated and work hard. John, however, is passive, dejected and seems to lack motivation. Did he display a vulnerability that normally was difficult to see because of his restlessness? Was John actively consolidating a position in which he did not participate alongside the others, because such a position was within his comfort zone? Brian describes John as “withdrawn” and “clearly bothered by something”, which shows an attention to John’s internal state and relates his participation to that state. The lack of symmetry in the boys’ positions points to a cooperative problem in the group, and might signal that John needs more, or a different kind of support than what is currently available to him. The rest of the group seem to have discovered their ability to cooperate and be creative. Paul shows himself as a skilled improviser:

*Marsha’s Diary Week 3, Day 2*: At the very end today, when we were supposed to be finished, Paul suggested that we sang *The Sea Is So Calm*. We sang the song, and during the verse about the sea being so calm Paul suddenly sang: “The sea is so calm, in the night, the sea is so calm all night long” instead of “The sea is so calm, so calm, so calm, the sea is so calm” which is the original version. It’s like he really feels the music, and does what comes to him musically.

(…) Before the *Face Song*, we invited Paul to improvise in front of the other children. He concentrated with great effort and played with his whole heart. It was so real and so good that the other children were actually quiet and enjoyed themselves. At one point they started clapping to the beat. (…) He was really proud when he was finished, and smiled from ear to ear during the *Face Song* afterwards.

Paul follows the shared structure, but finds room to improvise. His poetic suggestions increase the complexity of the song, intensifies the emotional tone and ambiance related to the ending of a session, and makes the song more interesting to sing. The music therapy students are able to offer him a space that is motivating, personal and shared. This change in position from fooling around to showing himself as a skilled improviser shows the others that he can engage in sustained and synchronized interaction. This position comes with an increased and more mature repertoire that broadens Paul’s possibilities for interaction. Based on Paul’s ability to improvise during these sessions, the students invite him to improvise live on-stage during the performance.

### Performing together

Marsha had been ill during the days running up to the performance. On the day of the first performance, she was well again and could re-join the group. Paul greeted her with “Marsha! How nice to see that you are well again!”

*Diary of PI Week 4, Day 2, Day of first performance*: Paul took quite a long time to start playing; it looked like he had a plan and couldn’t decide where to start. One of the students offered him a small recorder. Shortly after, she invited him to play on the bells. When none of these got him going, I offered him a set of drumsticks. One of the students tapped gently on the drum, and this was probably the cue he needed, because that seemed to get him going. He played elegantly on the small drum and had a bell in his left hand. During the dress rehearsals, he had used the shaker and several other instruments and played with more vigour. It is brave of him to improvise like this, but the children were patient and made it into a good session after all.

Paul’s improvisation is spontaneous, planned, and dependent on collaboration and timing. The students, the music therapist and the PI do their best to facilitate and support Paul’s performance. Paul plays with a narrower use of instruments and with less vigour than during rehearsals, but is still able to show his playfulness and musical talent. The other children in the group and the audience support him by being attentive, patient and by applauding at the end of his piece.

*Diary of Brian Week 4, Day 2, Day of first performance*: The boys really hit the mark! This time, John stood together with the other boys and sang. A couple of great “ooooii!”-moments from the audience when the boys stood on their heads and performed wonderfully! Towards the end they prepared to jump and 1 …2 …3! Done.

The boys are performing together, and John has finally fully joined the group and aligned his position with that of the others. He told the students the day before that he wanted to participate and sing in a microphone on the day of the concert, and not before. He kept his promise and did not sing during the dress rehearsal. This shows how John mindfully and with agency took a different role than he had chosen to or been able to before. What was the turning point for him? Perhaps it was a question of timing and ownership, which seemed to be affected by awareness of the upcoming performance. John used the performance to structure a change in his own participation towards being more active and self-assertive on stage. Marsha writes:

*Diary of Marsha Week 4, Day 2, Day of first performance*: John had said earlier that day to Klara that he wanted to sing this time, in a microphone. He did not do this during dress rehearsals. He seemed a bit reserved. He clearly let us know that he would not do it now, but he would do it at the performance. During the performance when it was time for his part, Brian forgot to give him the microphone. John stood markedly and steady, and asked loudly and clearly several times to get the microphone. It was a strange experience to see him so being so markedly and not “afraid of his own shadow”, when all the other times he has been so visibly unsure.

It is interesting how there is such a clear change in John’s participation. Despite overlapping descriptions by the three adult observers, John’s internal process remains a mystery. During the follow-up interviews, the PI asked him about the project, and John was very positive about the whole process. He started the interview by telling her that it was “a lot of fun to sing in the microphone”. He described the audience as “smiling and happy”, told her that he “sang his own song”, and that it was “fun that his best friends were there”. Unlike the other boys, no one from John’s family came to see him perform.

### Discovering ripple effects

By being in the audience, Paul’s parents were able to experience a different side of Paul, and get to experience him as skilled, independent and mature:

*Diary of PI Week 4, Day 2, Day of first performance*: We had a good chat with Paul’s parents. They got to hear about all the good impressions we have had of Paul and the good interaction we have experienced. We told them how talented Paul is, socially sensitive and attentive in his interactions with others, and that he seems to have a passion for entertaining others. The mother had tears in her eyes during our conversation; I think she was a bit overwhelmed by all the positive comments. (…) The mother said it was a big deal for her to see him on stage; it was unfamiliar and he looked very concentrated and mature. She was very happy, and so was the father.

The performance creates an opportunity to reflect on Paul’s performance with his parents and a staff member from the local pedagogical resource centre. Paul’s parents wanted him to continue music therapy, and this conversation revealed that the parents sensed a need for both structures and resources in the local environment. The mother was the most verbal about Paul’s process and change, or at least the PI reports her reactions and reflections with more emphasis than the father’s reactions and reflections.

The educational leaders revealed that they felt the project had been quite exhausting and more resource demanding then they had expected. Still they were very pleased with development of the boys and felt that this had “made it all worth it”. About a month after the end of the community music therapy project, the students visited the kindergarten to say goodbye to the children, and share a recording of the performance:

*Marsha’s Diary, One month after last performance*: When the break show started, George and Paul became very excited. They thought it was really cool. They didn’t say much, but paid close attention. (…) [When the film ended] some of the children ran out again quickly because they wanted to play. Paul stayed behind, together with two of the other children. Vera, the educational leader, asked if he was going to miss us. Paul said that he didn’t need to miss us, because he could watch the film every time he thought about us. That way, he was able to see us. And we could do the same if we missed them. He said that he would show the performance to his children, and that his children would get the DVD so that they could show it to their own children. During the film at one point, George said that it was boring to watch. Then Paul answered very clearly: “I think it is a lot of fun, myself!”

George and Paul’s excitement and the fact that they pay close attention to the film, shows ownership of the project, and indicates that it has been meaningful for them. Paul talked about the project and the performance in particular as if it was already a part of his identity and personal narrative. Paul even outlined how the project can have ripple effects on an ecological timeline into the future, as part of his personal legacy.

## Discussion

We now return to the research question to be discussed in this article: How can children’s restlessness be understood as a process? In order to lift the discussion beyond the particular context of community music therapy, and in order to relate it to a broader discussion about children’s restlessness and ADHD, we will use seven qualities that are seen as central to a community music therapy approach. Stige and Aarø (2012) have used the acronym PREPARE to describe these qualities: participatory, resource-oriented, ecological, performative, activist, reflective and ethics-driven. The specific series of seven qualities was generated as a synthesis of Stige’s (2002) theoretical study informed by sociocultural perspectives and eight ethnographic case studies of community music therapy practices in diverse cultures (Stige et al., 2010). We will now discuss these qualities by relating them to Paul, John, George and Ryan’s process, and to the case studies presented in the literature review.

### Reflecting on participatory and resource-oriented qualities

Children’s restlessness and the possibility of understanding restlessness as ADHD, was a topic for both the research project and for the kindergarten. The kindergarten worried about the boys’ behaviours as they transitioned into the school setting, and the research project was initially thought of as a possible preventative system intervention for children that would fit behaviours listed in the ADHD diagnosis. Central to the ADHD diagnosis is the individual child’s problems with attention, hyperactivity, and impulse control (American Psychiatric Association, 1980).

In line with this focus, although not in diagnostic terms, the PI, Brian, and Marsha also gave problem- and individual-oriented descriptions of the boys’ participation, especially during the phase *Exploring musical vitality and cooperation*: “the three noisy boys”, “not prepared to follow instructions”, “created a lot of unrest”, and “restless”. Other problem-oriented descriptions in this phase refer to difficulties with cooperation: “fooling around”, “the session was unstructured, noisy and chaotic”, “really out of hand today”, “all over the place”, and “things never fell into place”. However, there are also resource-oriented descriptions of their participation during this phase: “activated”, “eager”, and “cuddly”.

During the phases *Consolidating positions* and *Performing together*, the boys are described more in terms of resources and constructive participation: “Everyone made suggestions”, “A great start to a great day”, “concentrated with great effort and played with his whole heart”, “sweet”, “he played elegantly”, and “brave”. In other words: problem descriptions dominate the first phase of the project, but as positions start to consolidate and the focus becomes that of performing together, the descriptions become more resource-oriented and the children’s participation is described to a larger degree as well-functioning.

In line with a neurobiological perspective on ADHD (Barkley, 1997; Høvik & Plessen, 2010; Konrad & Eickhoff, 2010), this development would mean that neuro-developmental dysfunctions have miraculously been restored or that the situation has changed in such a way that the neurodevelopmental disorder has become more hidden and less dysfunctional. By looking at the same development from a community music therapy perspective, one could say that exploring a new territory together can be challenging, yet important in allowing children’s participation and in discovering their resources.

We have presented the results from our study as phases in order to show how such processes take time, how restlessness appears and disappears in context, and how there is meaning to this behaviour. If children’s restlessness is assessed on singular occasions, or assessed by persons in relation to whom they have consolidated dysfunctional roles or feel insecure, one is at risk of confirming problem-oriented stereotypes. Behaviour-oriented diagnoses like ADHD offer superficial, problem-focused and generalized descriptions.

The critical interpretive literature review shows that a dominating focus in music therapy research has been the individual child’s problematic behaviour, a focus that is compatible with a diagnostic approach. The diagnostic approach is also reflected in the fact that ADHD doubled in prevalence in North America between 1990 and 1995 (Neufeld & Foy, 2006) and is now estimated to have a world-pooled prevalence of 5.29% (Polanczyk et al., 2007). In Norway, which is the context for this study, hyperactivity, concentration problems, behavioural disturbances, and ADHD are presented as the most common reasons for referral to the mental health system (Mykletun, Knudsen, & Mathiesen, 2009).

Several of the music therapy case descriptions published after 1991 (Hibben, 1991; McFerran, 2009; Miller, 2011; Rickson, 2006) use the ADHD diagnosis to describe the child and in setting goals for therapy. However, these studies also contain descriptions of context and resources. The case descriptions from Europe (Achenbach, 2012; Hakomäki, 2012; Johns, 2012) do not include the ADHD diagnosis, but rather describe restlessness in relation to existential experiences, vitality, meaning, and group dynamics.

In summary, children’s participation, problems and resources are judged by adults on the basis of available observations and understandings. A diagnostic approach like ADHD offers understandings that emphasize individual- and problem-oriented aspects of children’s restlessness. The participatory- and resource-oriented qualities of music therapy offer complimenting and contrasting understandings. However, a focus on the individual and problematic aspects of John, Paul, George and Ryan’s participation is also central in the descriptions offered by the music therapy students and the PI. What is interesting is that these descriptions become less pronounced, and are supplied with descriptions of the boy’s resources and meaningful participation as the process unfolds. In this sense, a community music therapy perspective emphasizing participation and resources could be used to broaden, but also challenge, current mainstream understandings by pointing to the temporal, dynamic and contextual aspects of children’s restlessness.

### Reflecting on ecological and performative qualities

The kindergarten staff and the music therapy students sometimes experienced the restlessness that could arise when John, Paul, George and Ryan got together as challenging and resource demanding. Informed by a diagnostic perspective, the restlessness could be understood as hyperactivity, impulsivity or lack of concentration. Ultimately, such behaviour could be understood as symptoms of neurodevelopmental disorder that could become a serious dysfunction in a classroom setting. From another perspective, the restlessness could be understood as the performance of *relationships*. These relationships existed between the boys, and between the boys and other children and adults, but also between the boys and spaces, objects, and structures in the context. The totality of these relationships is referred to as *the ecological system* (Bronfenbrenner, 1979; Lerner, 2005). By offering the boys other qualities in these relationships, for instance by inviting them to participate in musical improvisation, John, Paul, George and Ryan had the opportunity to perform their relationships in new ways, with new people and with other objects. Working together towards a public performance, created structure and motivation. In contrast to an ADHD perspective, a community music therapy perspective can support a process-oriented contextualisation of the boys’ restlessness, and highlights the importance of cooperation within and between ecological systems.

Children diagnosed with ADHD in the case studies reviewed here are often boys in difficult life situations who struggle with social relationships and academic performance. Many of them are medicated for their behaviours or problems, and they live their life at the lower end of the socio-economic bracket. Working with the reciprocal relationships between individuals, groups, and networks in social context is central to the ecological quality of community music therapy (Stige & Aarø, 2012). Research on ADHD can also be used to point to the importance of understanding the child as a person developing in a certain context. For instance, family economy is a significant predictor of mental health problems (Bøe, Øverland, Lundervold, & Hysing, 2011). Furthermore, the aetiology of ADHD can be understood in context of socio-economic status, with parent attachment and/or family conflict as the mediating factor (Bøe et al., 2014; Russell, Ford, Rosenberg, & Kelly, 2013). It is also interesting that children assessed as at-risk at ages 5–6 years due to demographic factors and the display of behavioural, attention, academic or social problems, have similar academic scores and student–teacher relationships as their non-risk peers if placed in a strong instructional and supportive classroom during their first year of school (Hamre & Pianta, 2005). These examples point to the importance of regarding children’s restlessness as framed and influenced by qualities in their ecological systems.

In the literature presented here, the relationships between the children and their ecological systems are characterized by what can be understood as a lack of reciprocity. The main focus in many of the cases seems to be to relieve the child from suffering and to help the child make the best of the existing and unfortunate context. Beyond the literature presented here, it is clear that researchers, teachers, parents, health care professionals and policy makers can add to an exaggerated focus on behaviour, if they simply work towards reducing the externalization of restlessness in children. This is counteracted when researchers, teachers, parents, health care professionals and policy makers become curious about what the child is communication or trying to achieve. A qualified orientation towards the experiences of the child is referred to as *a child perspective* (Sommer, Samuelsson, & Hundeide, 2010).

There are examples of how music therapists can offer positive experiences for children that are regarded as restless, for instance by facilitating reciprocity. One example of this is when Will is given the opportunity to enjoy musical interaction with his mother and her therapist (Aigen, 1991), and when Ben (McFerran, 2009) and John (Rickson, 2003) are given the opportunities to receive validation and applause for their musical performances. In these cases, the music therapy context serves two potentially conflicting purposes: it is a space for validating self-expression and building reciprocal ecological relations, but it is also a context for reducing what is defined as negative behaviour in the systems surrounding the child. It might be, however, that negative behaviours are reduced because music offers a context and a medium through which experiences that are difficult to verbalize can be expressed. This aspect is for instance central in Hakomäki’s (2012) description of Nick’s process of creating meaning after losing his brother. Also, in McFerran’s case study of Ben, it is clear that music and music therapy offers both structure and freedom. Many of the case studies presented here show how a music therapy process can be unstructured and simultaneously highly facilitated.

In the literature review there are also descriptions of how the process of moving between ecological contexts can be a source of restlessness. For instance, McFerran (2009) describes how music therapy unintentionally adds stress to Ben’s life as he has to move back into the controlled setting of the classroom. McFerran describes how stimulant medication increases Ben’s realistic perception of himself and his abilities—a positive change from an educational perspective—but how it *simultaneously* hinders enjoyment and exploration; a negative change from a music therapy perspective. In other words, the stimulant medication *simultaneously* enhances and inhibits Ben’s ability to function. McFerran’s case study can serve to illustrate how children’s restlessness, like many other human phenomena, is paradoxical and contradictive. Reality is seldom a simple “yes” or “no”, just like the concept of “function” is seldom “functions well” or “is dysfunctional”. We all function well in some settings and not in others. Our function may vary with time of day, who we are with, and what we are doing. Dysfunction is often used to describe an individual, but reveals qualities in the context. If we are able to have a more nuanced view of children and how they function, we might find more constructive and humane ways of understanding and handling their restlessness.

### Reflecting on activist, reflective and ethics-driven qualities

During the community music therapy project, the music therapy students, the PI, the music therapists, the educational leaders, the children, and towards the end, the parents, exchanged perspectives on how the unfolding process could be understood. We used these reflections to discover ethical challenges and reflect on the activist qualities of our project. For instance, discovering that there was a lack of instruments, rooms, and infrastructures in the local community, prompted us to discuss this with parents, the kindergarten director, the head of the local school and representatives from the local pedagogical-psychological service.

We will now present an example of how triangulating the data from the three research diaries contributed to increased reflexivity regarding the (co-)constructed nature of not just the occurrence of behaviour during music therapy, but on the level of *observation* of that behaviour during music therapy.

The example contains two descriptions of the same situation. Marsha and the PI each describe their own efforts to scaffold Paul’s improvised performance. The PI writes:

One of the students offered him a small recorder. Shortly after, she invited him to play on the bells. When none of these got him going, I offered him set of drumsticks. One of the students tapped gently on the drum, and this was probably the cue he needed, because that seemed to get him going. He played elegantly on the small drum and had a bell in his left hand.

In an excerpt from Marsha’s diary not included in the results above, she writes:

He spent a lot of time finding something to play. At first it looked as if he was just going to sit there and move the instruments around and not get started. (…) Brian and I tried to help him into playing, and tried to get him started. But he did everything in his own pace and suddenly he started playing.

These two descriptions highlight the adults’ participation and provide two related, but different explanations of why Paul starts playing. Both the PI and Marsha seem to notice their own actions and efforts, and use these observations to explain Paul’s behaviour in different ways. Paul’s own perspectives and motives are hard, if not impossible, to observe directly in the moment, and therefore are given less attention. Both the PI and Marsha focus on their own efforts and intentions and overlook other possible explanations. As observers, we typically emphasize personal factors and overlook contextual factors, a tendency that provides us with a limited and self-centred understanding of a situation. This example can be taken as an illustration of how contextual reflections are difficult to make in the moment, which underlines the importance of a reflexive focus in supervision and research. Reflexive supervision can contribute to understanding one’s own emotional reactions and allow them to inform the research project (McFerran et al., 2016). Also, reflexive supervision can decrease defensive postures and facilitate constructive learning (Paré, Audet, Bailey, Caputo, & Hatch, 2004).

In the case studies in the literature review, ADHD, “lack of attention” or “hyperactive” are included as seemingly neutral concepts. Few explicitly critical voices were present in this research. However, Rickson and McFerran (2007) highlight the need for more in-depth and qualitative research on music therapy in special education, as this field traditionally has drawn heavily on behavioural principles. From a meta-perspective, the link between restless behaviours and the dysfunctional and marginalizing aspects of their ecological systems become evident. Some of the authors do indeed reflect on restlessness as a performance of ecological relationships, for instance Aigen (1991) who discusses Will’s problems as a function of difficult circumstances.

Reflecting on how an individual- and problem-oriented approach to children affects our understanding of children and their behaviour is not simply an epistemological or ontological question. It addresses a bigger and more subtle problem; do we recognize children as human beings? In classical child psychology, for instance in stage-models of development like that of Piaget (Piaget & Inhelder, 1969/2000), the child is presented as alone in the world, on his or her way toward adulthood—a “becoming”. A contrast to this is the view that is communicated in childhood sociology, where the child is viewed as a “being”, meaning a person and social actor (James, Jenks, & Prout, 1998, in Sommer et al., 2010). Recognizing the child as a person and social actor within a certain ecological system therefore means recognizing children as “human beings”, not simply “human becomings”.

Children’s rights to participate and be protected from harm are described in the Convention on the Rights of the Child (United Nations, 1989). These rights are central to a community music therapy approach. Facilitating children’s rights in practice, however, is a complex process. “Giving children voice” or “attending to unheard voices” is an ethical ideal in community music therapy (Stige & Aarø, 2012; Stige et al., 2010), but separating children’s voices from one’s own perspectives and experiences is challenging. According to a survey conducted in the USA, music therapists involved in the treatment of early elementary school children diagnosed with ADHD are generally happy with the outcomes of music therapy (Jackson, 2003). This can be interpreted in many ways, but reveals that systemic change is generally not considered an outcome of music therapy. This points to an ongoing and unsolved ethical dilemma in music therapy research on ADHD: we need to address the fact that many of these children struggle to survive in ecological systems that marginalize their participation.

### Conclusion

We have used a community music therapy perspective to explore restlessness as a process. We have presented a case study of four boys participating in a music therapy project and reflected on how children’s restlessness unfolds within certain relations and contexts as a co-constructed and dynamic phenomenon. We argue that this perspective can contrast and inform the problem- and individual-oriented understandings facilitated by an ADHD perspective. In the body of music therapy research presented here, ADHD is often used to describe the individual child’s problems, and is generally presented as a neutral or objective description of children’s restless behaviours. Using our own case description as an example, we have argued that community music therapy can be useful when working towards enhanced mutual relationships between the individual and his or her ecological systems. In contrast to individual- and problem-focused understandings, understandings that include participation, resources, ecology, performance, activism, reflexivity and ethics, can shed light on adult and systemic contributions to children’s restlessness.

## Notes

1. Norwegian folk tale figure that appears in a song called *Kjerringa med Staven* (*The old Woman with the Staff*), where she is described as a quaint and rural figure living in an isolated valley, surviving on her own.

## References

[CIT0001] Aasgaard T. (2005). Assisting children with malignant blood diseases to create and perform their own songs In Baker F. & Wigram T. (Eds.), *Songwriting: Methods, techniques and clinical applications for music therapy clinicians, educators and students* (pp. 1–15). London: Jessica Kingsley.

[CIT0002] Achenbach C. (2012). Nordoff-Robbins music therapy in a nursery setting In Tomlinson J., Derrington P., & Oldfield A. (Eds.), *Music therapy in schools. Working with children of all ages in mainstream and special education* (pp. 47–60). London: Jessica Kingsley.

[CIT0003] Aigen K. (1991). Creative fantasy, music and lyric improvisation with a gifted acting-out boy In Bruscia K. (Ed.), *Case studies in music therapy* (pp. 109–126). Phoenixville, PA: Barcelona.

[CIT0004] Alvesson M., & Sköldberg K. (2000). *Reflexive methodology*. London: SAGE Publications.

[CIT0005] American Psychiatric Association (1980). *Diagnostic and statistical manual of mental disorders* (3rd ed.). Arlington, VA: American Psychiatric Association.

[CIT0006] Ansdell G. (2010). Belonging through musicing: Explorations of musical community In Stige B., Pavlicevic M., Elefant C., & Ansdell G. (Eds.), *Where music helps: Community music therapy in action & reflection I* (pp. 41-64). Aldershot: Ashgate.

[CIT0007] Barkley R.A. (1997). Behavioral inhibition, sustained attention, and executive functions: Constructing a unifying theory of ADHD. *Psychological Bulletin*, 121(1), 65–94. 9000892 10.1037/0033-2909.121.1.65

[CIT0008] Barkley R. A. (2002). International consensus statement on adhd. *Clinical Child and Family Psychology Review*, 5(22), 89–111. 12093014 10.1023/a:1017494719205

[CIT0009] Bøe T., Øverland S., Lundervold A. J., & Hysing M. (2011). Socioeconomic status and children’s mental health: Results from the Bergen child study. *Social Psychiatry and Psychiatric Epidemiology*, 47(10), 1557–1566. 22183690 10.1007/s00127-011-0462-9

[CIT0010] Bøe T., Sivertsen B., Heiervang E., Goodman R., Lundervold A. J., & Hysing M. (2014). Socioeconomic status and child mental health: The role of parental emotional well-being and parenting practices. *Journal of Abnormal Child Psychology*, 42(5), 705–715. 24150864 10.1007/s10802-013-9818-9

[CIT0011] Bronfenbrenner U. (1979). *The ecology of human development: Experiments by nature and design*. Cambridge, MA: Harvard University Press.

[CIT0012] Bruscia K. E. (2014). *Defining music therapy.* (3rd ed.). University Park, IL: Barcelona.

[CIT0013] Burr V. (2015). *Social constructionism.* (3rd ed.). London: Routledge.

[CIT0014] Camilleri V. (2000). Music therapy groups: A path to social-emotional growth and academic success. *Educational Horizons*, 78(4), 184–189.

[CIT0015] Chong H. J., & Kim S. J. (2010). Education-oriented music therapy as an after-school program for students with emotional and behavioral problems. *Arts in Psychotherapy*, 37(3), 190–196. doi:10.1016/j.aip.2010.03.004

[CIT0016] Gold C., Voracek M., & Wigram T. (2004). Effects of music therapy for children and adolescents with psychopathology: A meta-analysis. *Journal of Child Psychology and Psychiatry*, 45(6), 1054–1063. 15257662 10.1111/j.1469-7610.2004.t01-1-00298.x

[CIT0017] Hakomäki H. (2012). Storycomposing as a method in music therapy. A collaborative experiment with a young co-researcher In Trondalen G. & Stensæth K. (Eds.), *Barn, musikk, helse* (pp. 147–171). Oslo: Norges Musikkhøyskole.

[CIT0018] Hamre B. K., & Pianta R. C. (2005). Can instructional and emotional support in the first-grade classroom make a difference for children at risk of school failure? *Child Development*, 76(5), 949–967. doi:10.1111/j.1467-8624.2005.00889.x 16149994

[CIT0019] Herman F. (1991). The boy that nobody wanted: Creative experiences for a boy with severe emotional problems In Bruscia K. E. (Ed.), *Collected work: Case studies in music therapy* (pp. 99–108). Phoenixville, PA: Barcelona.

[CIT0020] Hibben J. (1991). Group music therapy with a classroom of 6-8 year-old hyperactive-learning disabled children In Bruscia K. E. (Ed.), *Collected work: Case studies in music therapy* (pp. 175–189). Phoenixville, PA: Barcelona.

[CIT0021] Høvik M., & Plessen K. (2010). Emotional regulation and motivation in children with ADHD. *Tidssrift for Den Norske Lægeforening*, 130(23), 2349–2352. 10.4045/tidsskr.09.0121 21139659

[CIT0022] Jackson N. A. (2003). A survey of music therapy methods and their role in the treatment of early elementary school children with ADHD. *Journal of Music Therapy*, 40(4), 302–323. 15015907 10.1093/jmt/40.4.302

[CIT0023] James A., Jenks C., & Prout A. (1998). *Theorizing childhood*. Cambridge, UK: Polity.

[CIT0024] Johns U. T. (2012). Vitalitetsformer i musikk og kommunikasjon. [Forms of vitality in music and communication] In Trondalen G. & Stensæth K. (Eds.), *Barn, musikk, helse* (pp. 29–43). Oslo: Norges Musikkhøyskole.

[CIT0025] Kaslow F. W. (1996). *Handbook of relational diagnosis and dysfunctional family patterns*. New York, NY: Wiley.

[CIT0026] Kiesler D. J. (1991). Interpersonal methods of assessment and diagnosis In Snyder C. R. & Donelson R. F. (Eds.), *Handbook of social and clinical psychology* (pp. 438–468). New York, NY: Pergamon.

[CIT0027] Konrad K., & Eickhoff S. B. (2010). Is the ADHD brain wired differently? A review on structural and functional connectivity in attention deficit hyperactivity disorder. *Human Brain Mapping*, 31(6), 904–916. 20496381 10.1002/hbm.21058 PMC6871159

[CIT0028] Lerner R. M. (2005). Urie Bronfenbrenner: Career contributions of the consummate developmental scientist In Bronfenbrenner U. (Ed.), *Making human beings human. Bioecologial perspectives on human development* (pp. ix). London: SAGE Publications.

[CIT0029] Malloch S., & Trevarthen C. (Eds.). (2009). *Communicative musicality - Exploring the basis of human companionship*. Oxford: Oxford University Press.

[CIT0030] McCarty B. C., McElfresh C. T., Rice S. V., & Wilson S. J. (1978). The effect of contingent background music on inappropriate bus behavior. *Journal of Music Therapy*, 15(3), 150–156.

[CIT0031] McFerran K. (2009). Quenching a desire for power: The role of music therapy for adolescents with ADHD. *Australian Journal of Special Education*, 33(1), 72–83.

[CIT0032] McFerran K. S., Hense C., Medcalf L., Murphy M., & Fairchild R. (2016). Integrating emotions into the critical interpretive synthesis. *Qualitative Health Research*, 1(11). doi:10.1177/1049732316639284 27055499

[CIT0033] Miller E. B. (2007). Getting from psy-phy (psychophysiology) to medical policy via music and neurofeedback for ADHD children. *Dissertation Abstracts International: Section B: the Sciences and Engineering*, 68(3–B), 1977.

[CIT0034] Miller E. B. (2011). The brain-jamming for focus finale: Helping ADHD children with bio-guided music therapy. *Collected Work: Proceedings: 13. World Congress of Music Therapy. (AN: 2011-19799*), 9(1), 164–165.

[CIT0035] Moncrieff J., & Timimi S. (2013). The social and cultural construction of psychiatric knowledge: An analysis of NICE guidelines on depression and ADHD. *Anthropological Medicine*, 20(1), 59–71. doi:10.1080/13648470.2012.747591 PMC4095945 23496174

[CIT0036] Mykletun A., Knudsen A. K., & Mathiesen K. S. (2009). *Psykiske lidelser i Norge: Et folkehelseperspektiv*. [Mental health issues in Norway: A public health perspective]. Oslo, Norway: Norwegian Institute of Public Health [Folkehelseinstituttet].

[CIT0037] National Institutes of Health Consensus Development Conference Report (2000). Diagnosis and treatment of attention deficit hyperactivity disorder (ADHD). *Journal of Pharmaceutical Care in Pain & Symptom Control*, 8(3), 75–89.

[CIT0038] Neufeld P., & Foy M. (2006). Historical reflections on the ascendancy of ADHD in North America, c. 1980 - c. 2005. *British Journal of Educational Studies*, 54(4), 449–470.

[CIT0039] Olsvold A. (2012). *Når “ADHD” kommer inn døren: En psykososial undersøkelse av barns, mødres og fedres forståelse og opplevelse av ADHD-diagnose og -medisinering*. [When “ADHD” enters: A psychosocial investigation of children’s, mother’s and father’s understanding and experience of ADHD diagnosis and medication] (PhD Thesis). Oslo, Norway: University of Oslo.

[CIT0040] Paré D., Audet C., Bailey J., Caputo A., & Hatch K. (2004). Corageous practice: Tales from reflexive supervision. *Canadian Journal of Counselling*, 38(2), 118–130.

[CIT0041] Pavlicevic M., & Ansdell G. (2004). *Community music therapy*. London: Jessica Kingsley.

[CIT0042] Piaget J., & Inhelder B. (1969/2000). *The psychology of the child*. New York, NY: Basic Books.

[CIT0043] Polanczyk G., Lima M. S. D., Horta B. L., Biederman J., & Rohde L. A. (2007). The worldwide prevalence of ADHD: A systematic review and metaregression analysis. *The American Journal of Psychiatry*, 164(6), 942–948. 17541055 10.1176/ajp.2007.164.6.942

[CIT0044] Polanczyk G., Willcutt E. G., Salum G. A., Kieling C., & Rohde L. A. (2014). ADHD prevalence estimates across three decades: An updated systematic review and meta-regression analysis. *International Journal of Epidemiology*, 43(2), 434–442. 24464188 10.1093/ije/dyt261 PMC4817588

[CIT0045] Rickson D. J. (2002). Adam: A case study of an adolescent boy. *Annual Journal of the New Zealand Society for Music Therapy*, 51–60.

[CIT0046] Rickson D. J. (2003). The boy with the glass flute. *Voices: A World Forum for Music Therapy*, 3(2). doi:10.15845/voices.v3i2.124

[CIT0047] Rickson D. J. (2006). Instructional and improvisational models of music therapy with adolescents who have attention deficit hyperactivity disorder (ADHD): A comparison of the effects on motor impulsivity. *Journal of Music Therapy*, 43(1), 39–62. 16671837 10.1093/jmt/43.1.39

[CIT0048] Rickson D. J., & McFerran K. (2007). Music therapy in special education: Where are we now? *Kairaranga*, 8(1), 40–47.

[CIT0049] Rowland A. S., Lesesne C. A., & Abramowitz A. J. (2002). The epidemiology of attention-deficit/hyperactivity disorder (ADHD): A public health view. *Mental Retardation and Developmental Disabilities Research Reviews*, 8, 162–170. 12216060 10.1002/mrdd.10036

[CIT0050] Russell G., Ford T., Rosenberg R., & Kelly S. (2013). The association of attention deficit hyperactivity disorder with socioeconomic disadvantage: Alternative explanations and evidence. *The Journal of Child Psychology and Psychiatry*, 55(5), 436–445. 24274762 10.1111/jcpp.12170 PMC4263245

[CIT0051] Ruud E. (1998). *Music therapy: Improvisation, communication and culture*. Gilsum, NH: Barcelona.

[CIT0052] Sausser S., & Waller R. J. (2006). A model for music therapy with students with emotional and behavioral disorders. *The Arts in Psychotherapy*, 33(1), 1–10.

[CIT0053] Singh I. (2004). Doing their jobs: Mothering with Ritalin in a culture of mother-blame. *Social Science & Medicine*, 59(6), 1193–1205. doi:10.1016/j.socscimed.2004.01.011 15210091

[CIT0054] Small C. (1998). *Musicking: The meanings of performing and listening*. Middletown, CT: Wesleyan University Press.

[CIT0055] Sommer D., Samuelsson I. P., & Hundeide K. (2010). *Child perspectives and childrens’ perspectives in theory and practice*. Dordrecht Heidelberg London New York: Springer.

[CIT0056] Stake R. E. (1994). Case studies In Denzin N. K. & Lincoln Y. S. (Eds.), *The SAGE handbook of qualitative research*. London: Sage.

[CIT0057] Steele A. L., Vaughan M., & Dolan C. (1976). The school support program: Music therapy for adjustment problems in elementary schools. *Journal of Music Therapy*, 13(2), 87–100.

[CIT0058] Stern D. N. (2010). *Forms of vitality: Exploring dynamic experience in psychology, the arts, psychotherapy, and development*. Oxford: Oxford University Press.

[CIT0059] Stige B. (2002). *Culture-centered music therapy*. Gilsum, NH: Barcelona Publishers.

[CIT0060] Stige B., & Aarø L. E. (2012). *Invitation to community music therapy*. New York, NY: Routledge.

[CIT0061] Stige B., Ansdell G., Elefant C., & Pavlicevic M. (2010). *Where music helps: Community music therapy in action and reflection*. Surrey: Ashgate.

[CIT0062] Subcommittee on Attention-Deficit/Hyperactivity, D., & Committee on Quality, I (2001). Clinical practice guideline: Treatment of the school-aged child with attention-deficit/hyperactivity disorder. *Pediatrics*, 108(4), 1033–1044. doi:10.1542/peds.108.4.1033 11581465

[CIT0063] Timimi S. (2005). *Naughty boys: Anti-social behaviour, ADHD and the role of culture*. Hampshire: Palgrave McMillan.

[CIT0064] Timimi S., & Taylor E. (2004). ADHD is best understood as a cultural construct. *British Journal of Psychiatry*, 184, 8–9. 14702221 10.1192/bjp.184.1.8

[CIT0065] Trevarthen C. (2000). Musicality and the intrinsic motive pulse: Evidence from human psychobiology and infant communication. *Musicae Scientiae*, 3(1 suppl), 155–215. doi:10.1177/10298649000030s109

[CIT0066] Ullebø A. K. (2010). *Epidemiology of ADHD. Screening, prevalence and phenomenology of the Attention deficit/Hyperactivity Disorder phenotype* (PhD Thesis). University of Bergen, Bergen.

[CIT0067] UN Convention on the Rights of the Child: Adopted and opened for signature, ratification and accession by General Assembly resolution 44/25 of 20 November 1989 entry into force 2 September 1990, in accordance with article 49. (1989).

[CIT0068] Willig C. (2008). *Introducing qualitative research in psychology.* (2nd ed.). Berkshire, UK: McGraw Hilll Open University Press.

